# Three-Dimensional Spheroid Culture of Human Mesenchymal Stem Cells: Offering Therapeutic Advantages and In Vitro Glimpses of the In Vivo State

**DOI:** 10.1093/stcltm/szad011

**Published:** 2023-05-15

**Authors:** B Linju Yen, Chen-Chan Hsieh, Pei-Ju Hsu, Chia-Chi Chang, Li-Tzu Wang, Men-Luh Yen

**Affiliations:** Regenerative Medicine Research Group, Institute of Cellular and System Medicine, National Health Research Institutes (NHRI), Zhunan, Taiwan; Regenerative Medicine Research Group, Institute of Cellular and System Medicine, National Health Research Institutes (NHRI), Zhunan, Taiwan; Institute of Molecular Medicine, National Tsing Hua University, Hsinchu, Taiwan; Regenerative Medicine Research Group, Institute of Cellular and System Medicine, National Health Research Institutes (NHRI), Zhunan, Taiwan; Regenerative Medicine Research Group, Institute of Cellular and System Medicine, National Health Research Institutes (NHRI), Zhunan, Taiwan; Graduate Institute of Life Sciences, National Defense Medical Center (NDMC), Taipei, Taiwan; Department of Obstetrics and Gynecology, National Taiwan University (NTU) Hospital & College of Medicine, NTU, Taipei, Taiwan; Department of Obstetrics and Gynecology, National Taiwan University (NTU) Hospital & College of Medicine, NTU, Taipei, Taiwan

**Keywords:** 3-dimensional cell culture, multicellular spheroid, human mesenchymal stem cells, multilineage differentiation, chondrogenesis, osteogenesis, paracrine factors, immunomodulation, angiogenesis, wound-healing

## Abstract

As invaluable as the standard 2-dimensional (2D) monolayer in vitro cell culture system has been, there is increasing evidence that 3-dimensional (3D) non-adherent conditions are more relevant to the in vivo condition. While one of the criteria for human mesenchymal stem cells (MSCs) has been in vitro plastic adherence, such 2D culture conditions are not representative of in vivo cell-cell and cell-extracellular matrix (ECM) interactions, which may be especially important for this progenitor/stem cell of skeletal and connective tissues. The 3D spheroid, a multicellular aggregate formed under non-adherent 3D in vitro conditions, may be particularly suited as an in vitro method to better understand MSC physiological processes, since expression of ECM and other adhesion proteins are upregulated in such a cell culture system. First used in embryonic stem cell in vitro culture to recapitulate in vivo developmental processes, 3D spheroid culture has grown in popularity as an in vitro method to mimic the 3-dimensionality of the native niche for MSCs within tissues/organs. In this review, we discuss the relevance of the 3D spheroid culture for understanding MSC biology, summarize the biological outcomes reported in the literature based on such this culture condition, as well as contemplate limitations and future considerations in this rapidly evolving and exciting area.

## Graphical Abstract

**Figure:**
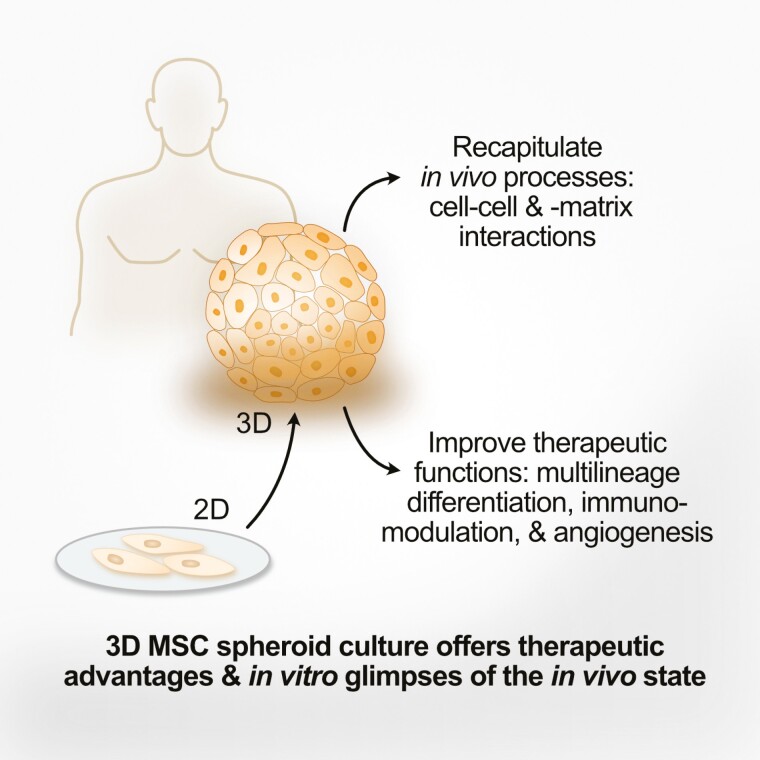

**Significance Statement**

While 2-dimensional in vitro plastic adherence is a criterion for defining human mesenchymal stem cells (MSCs), 3-dimensional (3D) non-adherent conditions may be particularly suited to understand MSC physiological processes since cell-cell and cell-extracellular matrix interactions in this system better mimic the native in vivo niche of MSCs within tissues/organs. Moreover, significant therapeutic advantages of 3D MSC spheroids have been seen, including in in vivo models. We discuss here the application of 3D spheroid culture for a better understanding of MSC in vivo biology and therapeutic uses, as well as limitations and future considerations in this rapidly evolving and exciting area.

## Introduction

In vitro mammalian cell culture has clearly been indispensable for understanding normal and pathological biological processes. As invaluable as this classic 2-dimensional (2D) monolayer system has been, the inability to mimic cell-cell and cell-extracellular matrix (ECM) interactions of cells within its native organs/tissues—all 3-dimensional (3D) in nature—are known to limit the physiological relevance of monolayer in vitro culture.^1-3^ Early morphologic studies demonstrate that when epithelial cells are cultured in 3D conditions, recapitulation into their in vivo native 3D structures mimicking its tissue of origin occurs.^4^ In recent years, profiling technologies have increasingly documented the genetic and epigenetic alterations that 2D in vitro culture can induce in the cultured cell, which then lead to genetic/chromosomal aberrations and functional phenotypes that drift away from the original state of the isolated cell.^5-8^ Alarmingly, a consistent difference reported between 2D monolayer culture and native tissue and/or uncultured cells is the upregulation of the cell cycle and proliferative pathways,^5,6^ which is often a key experimental parameter in most in vitro studies. Thus, while 2D monolayer cell culture is convenient and low cost, its physiological relevance is increasingly being questioned.

## Historical Background: 3D In Vitro Culture Recapitulate Physiological Outcomes With Cell-Type Specific Results

The earliest non-adherent 3D spheroids were likely spontaneous cell aggregations of differentiating pluripotent mouse teratocarcinoma cells grown in the absence of feeder cells to maintain undifferentiated conditions.^9^ Termed embryoid bodies (EBs), these suspended cell aggregates resembled rounded spheroids and, more importantly, proceeded to recapitulate the tri-germ layer differentiation events which occur during early mouse embryo development. When normal pluripotent stem cells (PSCs) were first isolated from the murine blastocysts, the same spontaneous aggregation of isolated embryonic stem cells (ESCs) into non-adherent EBs could also be seen when cultured without feeder cells to allow for spontaneous differentiation.^10^ 3D EB culture is now an established protocol to test the pluripotent capacity of any PSC including induced PSCs (iPSCs)^11,12^ and especially human PSCs,^13,14^ since the most rigorous tests of in vivo pluripotency for human cells are likely not able to be performed due to ethical concerns.^15,16^

In contrast to PSCs in which 3D suspension culture results in differentiation and developmental progression, stem cells from a number of adult organs including neural stem cells (NSCs) and mammary stem cells, are selected and identified through this same ability to survive and proliferate ex vivo from single-cells into suspended cellular spheroids in serum-free non-adherent culture.^17,18^ There are key differences between 3D conditions for PSCs versus somatic stem cells, however;3D PSC-EBs arise from aggregations of cells cultured in serum conditions, whereas the somatic stem cell spheroids arise from single-cell outgrowths and are cultured in serum-free conditions. This single-cell, minimal condition used to select for normal NSCs was then adopted to select for and identify cancer stem cells from solid tumors in the brain.^19^ Oddly, this same 3D NSC/brain cancer stem cell selection method has since been used to select for cancer stem cells from other solid cancers, including breast, colon, and lung.^20-22^ Indeed, there is still controversy in the idea of the “cancer stem cell” in solid tumors,^23,24^ and it is still of debate how reflective this stringent 3D culture method is to the actual disease state in the patient.^25^ These striking differences in cellular fate and developmental outcome after the 3D culture are clearly due to both differences in the culture method as well as the inherent nature of the cultured cell itself.

## 3D Spheroid Culture for “2D-Defined” MSCs: Evidence for In Vivo Relevance

MSCs are multipotent somatic stem cells that can differentiate into the mesodermal skeletal/connective tissue cell types of osteoblasts, chondrocytes, and adipocytes. First found in the bone marrow (BM) as supporting stromal cells for hematopoiesis, the multilineage differentiation capacity for these stem cells was rapidly demonstrated, with similar progenitor/stem cells also found in numerous tissues and organs in quick succession.^26^ An interesting defining characteristic of all MSCs is the in vitro criteria of plastic adherence in standard 2D culture.^27^ This unusual criterion likely arose out of the initial need in BM aspirates to discern MSCs from hematopoietic/immune cells which can adhere (ie, monocytes, macrophages, dendritic cells, and activated lymphocytes^28,29^) prior to the advent of more sophisticated molecular selection methods. It can therefore be argued that MSCs are the ultimate adherent cell type, so a fair question to ask would be whether 3D non-adherent culture conditions are an appropriate in vitro condition for MSCs. While the answer to this question still awaits accumulation of comparative evidence of in vivo vs. in vitro MSC biological information, there has been an explosion of publications on MSCs cultured in 3D conditions as spheroids: a PubMed search for title keywords of “mesenchymal stem/stromal cells,” “sphere” or “spheroid,” and “3D” yields nearly 300 publications. Scaffold-based systems of 3D MSC culture have a long history of study since the most important differentiation lineages of MSCs are toward skeletal-related tissues in which non-cellular components are functionally critical. Despite large numbers of studies in this area of 3D MSC culture and tissue engineering, shortcomings still exist in each type of scaffold, including effectiveness in mimicking the native ECM microenvironment, residual harmful solvent, uneven cell distribution, and maintenance of cell viability.^30,31^ While scaffold-based culture can activate MSC-matrix interactions, scaffold-free 3D spheroid culture relies on the cultured cells themselves to modulate and/or create the microenvironment as well as interact with each other; this is likely more physiological, and since this is a more recent 3D method, studies are generating unexpected and interesting findings.^32-34^ Importantly, the translational relevance of scaffold-free 3D MSC spheroids is increasingly being reported, with a very recent study evaluating detailed clinically important parameters in non-human primates.^35^ It is also necessary to clarify that in vitro culture of MSCs in scaffolds to achieve tissue engineering for 3D skeletal components like bone and cartilage still mainly involves the 2D adherent culture of MSCs on the engineered surfaces, and therefore not focused upon in this review.

Despite in vitro, plastic-adherence being one of the 3 criteria for defining human MSCs, in vivo these cells are obviously found in 3D tissues/organs.^36^ Indeed, the ability of MSCs to “self-assemble” into cellular aggregates and spheroids in suspension culture was reported in several studies shortly after the publication of the Minimal Criteria.^37-39^ Moreover, as the stem/progenitor cells for bone and cartilage, tissues where acellular components significantly outweigh the cellular compartment, MSCs are responsible for secreting the myriad of ECM molecules specific for each lineage.^40^ The multicellular 3D spheroid culture, therefore, may be particularly suited as an in vitro model to investigate MSC biology, as this method of 3D culture is known to elicit ECM secretion from the aggregated cells.^41^ In fact, an early study using bone progenitor cells/osteoblasts from diverse tissues including the BM demonstrated spontaneous non-adherent spheroid formation with osteogenic lineage commitment by all these MSC-related cells, with the spontaneous 3D spheroid formation increasing protein expression of integrins and inorganic components allowing for recapitulation of osteogenesis and successful ex vivo bone formation.^42^

As with its predecessor the EB suspension culture, the culture medium used for the multicellular 3D spheroid culture is usually unchanged from its 2D culture; this may be one important reason why this multicellular aggregated 3D culture method has been found to better reflect in vivo conditions, even for cancer cells.^41,43^ MSCs may be especially sensitive to its microenvironment both in vivo and in vitro, with specific ECM molecules, matrix, as well as the stiffness of its niche able to modulate differentiation and lineage commitment, as seen in the spontaneous differentiation into different lineages when cultured in vitro on plates with varying stiffness index and ECM protein coatings.^44,45^ The single-cell serum-free 3D culture may, therefore, be a less suitable method to recapitulate MSC biology given its minimal and stringent conditions; it has been well documented in a standard 2D culture that such low serum, low cell density conditions strikingly alter the biological profile of the cultured cell as to call into question the physiologic relevance of such methods.^46-48^ While advances in single-cell transcriptomic technology have been increasingly applied to understanding murine BM and adipose MSC in its native in vivo state,^49-51^ these studies are technically and ethically challenging to conduct in the human system. For understanding human MSCs in a more physiological context, researchers have therefore increasingly turned to use multicellular 3D suspension culture to achieve this goal.

## 3D Culture Is Integral to MSC Chondrogenesis and May Improve Osteogenic Differentiation as well as Multilineage and Survival Capacity

From the understanding of embryonic limb development elucidated in the 1980s-1990s, the standard protocol to induce MSC chondrogenesis in vitro has required 3D suspension culture conditions to achieve high-density cell aggregation, in contrast to differentiation protocols for all other somatic lineages which are largely performed using standard 2D monolayer culture. This is likely because condensation—a process during developmental lineage commitment in which reduced intercellular spaces, increased cell-cell adhesion, and increased ECM secretion lead to 3D tissue formation—is an integral process of in vivo chondrogenesis.^52,53^ Moreover, Sox9, the master transcription factor for chondrogenesis, can be induced by the process of compression and with 3D organoid culture.^54-56^ While the initial protocol for MSC chondrogenic differentiation protocol is similar to the typical 3D multicellular aggregation culture, one important difference is that chondrogenic differentiation requires a serum-free environment for adequate lineage commitment,^54^ but the influence of physical parameters brought about by 3D culture on MSCs to commit to chondrogenesis is so strong that this can occur even without the addition of biochemical factors including transforming growth factor β (TGFβ), the standard for this protocol.^40^

In contrast to MSC chondrogenesis where 3D multicellular non-adherent culture is a prerequisite, 3D culture to explore other functional capabilities of MSCs was first attempted in the late 2000s. A frequent result after MSC 3D spheroid formation is increased expression of pluripotency factors Oct4, Sox2, and Nanog,^57-59^ but the functional role of these findings with regard to MSCs is still of some debate.^60-62^ Multilineage differentiation capacity in terms of both adipogenic and osteogenic potential was also found to be increased in numerous studies;^58,59,63,64^ but strikingly, none of these reports evaluated chondrogenic potential, perhaps because studies were performed in serum-containing conditions. In contrast, one study found just chondrogenic capacity to be enhanced after 3D spheroid formation.^57^ Of the differentiation capacity found to be enhanced after 3D spheroid formation in serum conditions, increased osteogenesis has been most frequently documented in both in vitro and in vivo models,^65-67^ with one recent study demonstrating 3D spheroid MSCs could be rapidly induced into osteocytes, an even more mature cell type than osteoblasts.^68^ The strong osteogenic propensity of serum-cultured 3D MSCs is such that, in the few 3D MSC studies using non-human cells, similar results were found as well.^69,70^ 3D culture profoundly changes cell shape and cytoskeletal dynamics, and these parameters are known to influence MSC lineage commitment especially osteogenesis;^71^ in fact, spontaneous osteogenesis can occur when MSCs are cultured in 3D microcarriers as a result of cytoskeletal alterations.^72^ Also, similar to the early report on MSC-related osteoprogenitors enhancing osteogenesis after spontaneous spheroid formation,^42^ these MSC 3D studies were carried out in serum-containing conditions, implicating the importance of serum on influencing MSC commitment into osteoblasts versus chondrocytes.^73^ Increased protein expression of ECM molecules,^69^ osteogenic integrins,^67^ and cadherins^66^ were all found to be involved in the enhanced osteogenic capacity of 3D MSC spheroids. Such results further support that the 3D spheroid culture can recapitulate aspects of in vivo ECM/niche-cell and cell-cell adhesion and interactions less evident in 2D monolayer culture.^39,41,74,75^

One of the most consistent findings when MSCs are cultured as 3D spheroids appear to be increased cell viability and survival.^76^ This was very comprehensively evaluated in a report which not only assessed MSC spheroids but also human ESC spheroids.^77^ The enhanced cell survival can be seen across different culture conditions, including in minimal or serum-free conditions^39,58,78^ as well as hypoxia.^79,80^ Most studies found that MSCs cultured as 3D spheroids remain in a more quiescent, less proliferative state compared to 2D monolayer culture due to metabolic shifts and deregulation of cell cycle genes.^74,77,81^ While one report found increased apoptosis with 3D spheroid formation,^82^ nearly all other reports showed either maintenance of cell viability or decreased apoptosis in vitro as well as after in vivo transplantation.^58,59,79,80,83^ A few studies have delved into the mechanisms involved and identified the upregulation of anti-apoptotic genes such as Bcl-2 and the downregulation of apoptotic genes like Bax in MSC 3D spheroids.^77,84^ Collectively, these results implicate the higher relevance of 3D culture to native in vivo states/uncultured cells which by transcriptomic and epigenetic profiling analyses are less proliferative and more quiescent than 2D monolayer-cultured cells.^5^

## 3D MSC Culture Enhance Immunomodulation, Angiogenesis, and Paracrine Activities

While not an essential criterion, the strong immunomodulatory properties of nearly all sources of human MSCs have become one of the most clinically relevant functions of these stem/progenitor cells, by not only broadening the application of MSCs toward immune and inflammatory diseases but also allowing for immunologically unmatched use of these cells as off-the-shelf products.^85^ Interestingly, among the first reports to explore 3D MSC culture and functional outcomes was focused on immunomodulation.^81^ More in-depth research by the same group demonstrated specific mechanisms involved in the increased anti-inflammatory and immunomodulatory effects of 3D spheroid MSCs, which included triggering of caspase-dependent interleukin (IL)-1 signaling and the secretion of major immunomodulatory factors including prostaglandin E_2_ (PGE_2_) and tumor necrosis factor-stimulated gene 6 (TSG6),^86^ as well as suppression of inflammatory cytokines such as tumor necrosis factor-α (TNFα).^81^ The stronger immunomodulatory function of 3D cultured-MSC spheroids has been quite consistently reported,^58,87,88^ and compared to 2D culture, priming MSC spheroids with inflammatory factors such as interferon-γ (IFNγ)^89,90^ or IL-1^76,91^ also leads to increased immunomodulatory effects in vitro. In vivo, 3D spheroid MSCs led to better resolution of murine peritonitis^81^ and colitis.^77^ Moreover, 2 recent studies focusing on different types of arthritis have demonstrated better outcome with 3D spheroid MSCs: (1) in a murine rheumatoid arthritis model, just injection of the conditioned medium of 3D MSC spheroids resulted in a better outcomes than the application of the cells themselves whether 2D- or 3D-cultured;^92^ and (2) in a non-human primate model of osteoarthritis, injection of either xenogeneic or allogeneic 3D MSC spheroids decreased joint inflammation and improved disease outcome.^93^ While functional improvement of MSC immunomodulation was clearly demonstrated in all these studies, mechanistic understanding of how cell dimensionality can alter immune function awaits further elucidation.

Similar to immunomodulation, the angiogenic and wound-healing capacity of MSCs are well reported despite not being one of the Minimal Criteria. Paracrine factors are also largely responsible for many of these therapeutic effects and even in the first reports, increased vascular endothelial growth factor (VEGF) secretion after 3D spheroid culture was consistently seen to enhance these properties;^37,75,79,80,84,94,95^ other angiogenic and/or mitogenic factors especially hepatocyte growth factor (HGF) and basic fibroblast growth factor (bFGF) were also frequently reported to be upregulated.^37,74,79,84,94,95^ A number of in vivo ischemic disease models demonstrated therapeutic effects of 3D MSC transplantation, including in ischemic limb injury,^79,84^ ischemic kidney injury,^75^ and ischemic heart disease.^96^ There are also many studies showing 3D MSC spheroid application improving wound healing and involving angiogenesis in mouse models.^74,83,95^ Mechanistically, this appears to be due to the strong upregulation of hypoxia-related pathways in 3D MSC spheroids as evidenced by transcriptomic profiling,^37^ as hypoxia is one of the strongest inducers of angiogenesis.^97^ Collectively, these studies and immune-related studies demonstrate that, compared to 2D monolayer culture, 3D spheroid culture further enhances the paracrine functions of MSCs to have strong translational value.

## 3D MSC Culture Modulates ECM Molecules: Implications for Lineage Commitment and Stemness/Senescence

**Figure 1.**
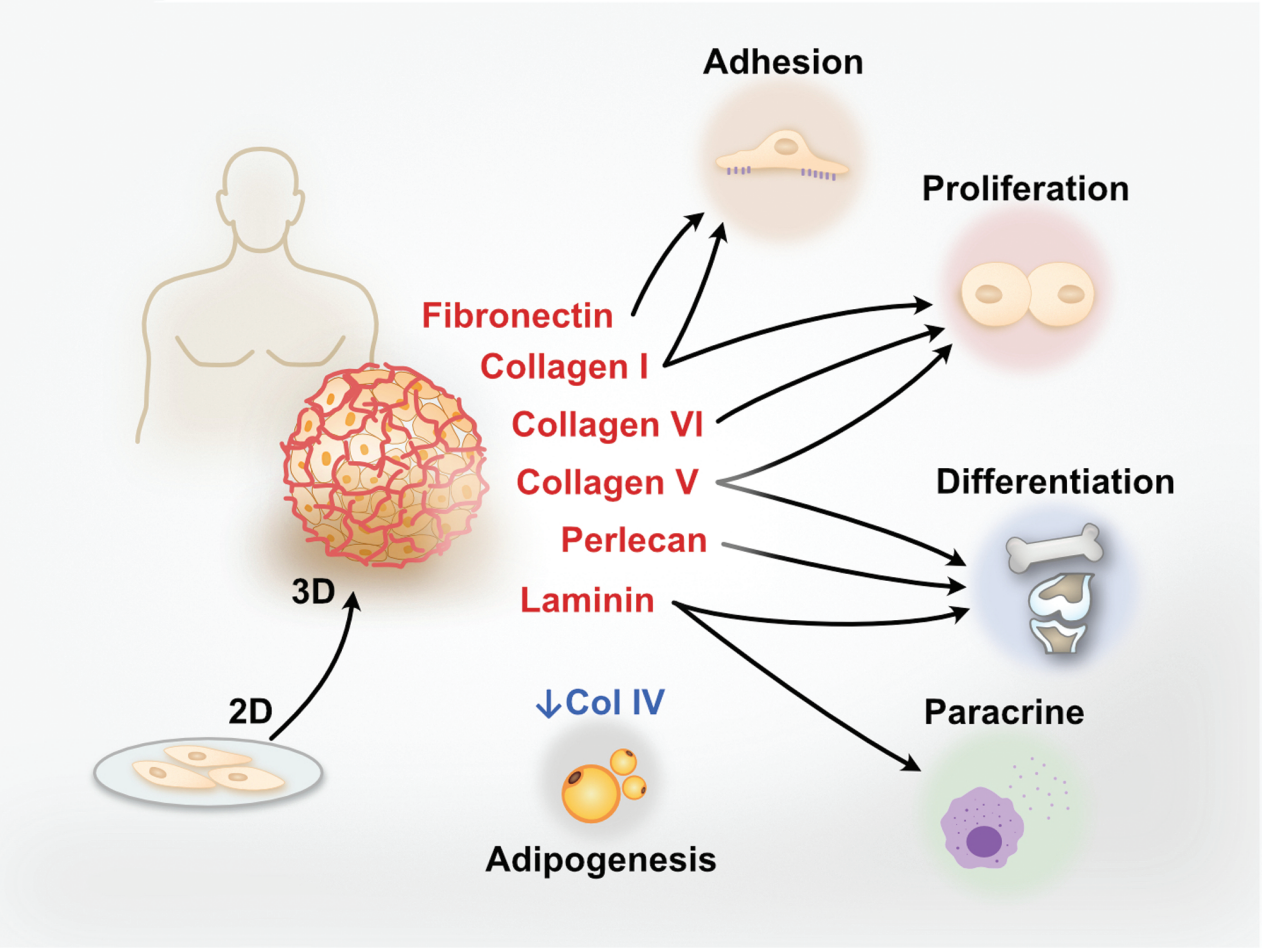
Effects and expression of ECM molecules in 3D-cultured MSC spheroids. MSCs are known to secrete a number of ECM molecules which can regulate stem cell fate. Compared to 2D monolayer system, ECM molecules including collagen I, IV, V VI, as well as fibronectin, laminin, and perlecan have been reported upregulated or highly expressed in human MSC 3D spheroids. The effects of these ECM molecules on MSCs examined through 2D monolayer model can be categorized to enhance cell adhesion, proliferation, differentiation, and paracrine effects according to previous research. In contrast, collagen IV, which upregulated during adipogenic differentiation, was found less expressed in MSC spheroids.

A number of studies have shown that the ECM can regulate stem cell fate, especially in MSCs.^98^ Conversely, MSCs are known to secrete a number of ECM molecules and remodeling enzymes due to the capacity of these stem cells to differentiate into tissues with significant ECM components. While such studies are beginning to be conducted using 3D-cultured MSC spheroids, most reports especially report including in vivo evaluation have largely used standard 2D culture systems; we have therefore summarized 2D-cultured MSC-ECM molecule studies along with 3D spheroid studies to better present the potential functional application of 3D MSC spheroid culture in modulating ECM molecules (Supplementary Table S1; Fig. 1). Upregulation of collagen I, fibronectin 1, and laminin were observed in 3D spheroid compared to 2D monolayer culture, and all 3 molecules are involved in increasing survival, proliferation, paracrine effects as well as stem cell selection/enrichment of MSCs.^75^ Collagen V and collagen VI were also highly expressed in MSC spheroids and reported to enhance proliferation.^95^ These results indicate that the enhancement of MSC stem cell properties by 3D spheroid culture could contribute to the expression of specific ECM components including collagens I, V, VI, as well as fibronectin and laminin. Some ECM molecules reported to modulate MSC differentiation are also observed in MSC spheroids. Upregulation of collagen V,^99^ laminin,^100^ and perlecan^101^ was seen in MSC spheroids; in 2D studies, these ECM molecules respectively were seen to promote chondrogenesis, neurite outgrowth, and osteogenesis while blocking adipogenesis. Interestingly, collagen IV, which in 2D studies is upregulated during adipogenic induction, is less expressed in 3D MSC spheroid cultures.^102^ Since it has been reported that senescent MSCs activate adipogenesis but suppress osteogenesis,^103^ the lower expression level of collagen IV in 3D MSC spheroids is in line with the ability to maintain stemness and avoiding senescence.

## Disease-Specific 3D MSC Spheroid In Vivo Studies: Recent Advances and Implications for Therapeutic Applications

Because culturing MSCs in 3D spheroid conditions is a newer methodology, there are much fewer studies in general compared to studies using standard 2D culture methods (Supplementary Table S2). However, in the relatively smaller pool of in vivo disease model studies using 3D MSC spheroid cultures, a surprisingly large proportion have focused on the differentiation capacity towards osteogenesis in the repair of bony defects using rodent calvarial defect models^65,70,104^ and femur fracture model;^88^ one recent study specifically evaluated 3D MSC spheroids for use toward calvarial defects in aged mice,^105^ which is in line with the overall in vitro finding of increased stemness/decreased senescence of 3D-cultured MSCs. A recent study evaluated the use of matrilin-3-primed MSC spheroids in a rat model of intervertebral disc degeneration.^106^ Also recently, secretome from 3D MSC spheroids have also been found to improve a mouse model of rheumatoid arthritis;^92^ more significantly and also very recently in a non-human primate model of osteoarthritis, direct intra-articular injection of either human BM- or ESC-MSC spheroids have also demonstrated therapeutic improvement.^93^ In these joint-related studies, some of the therapeutic effects could be attributed to immunomodulation since inflammation is a known component of any type of arthritis.

A significant number of in vivo studies have focused on paracrine properties of 3D MSC spheroids, with the earliest study revealing enhanced immunomodulatory properties in a mouse model of peritonitis.^81^ Subsequently, others have found similar results in a mouse model of colitis,^77^ and more recently in a mouse model of pulmonary inflammation.^107^ Another important MSC paracrine function is angiogenesis, and 3D spheroid administration has been evaluated in several rodent limb ischemia models.^79,84,108^ Wound-healing is a related angiogenic outcome and has been studied using either healthy^83,95^ or diabetic rodent models.^74^ Other organ-ischemia models likely benefitting from both enhanced angiogenic and immunomodulatory functions in addition to possible differentiation effects of 3D MSC spheroids include acute kidney injury in rats^75^ and a large-animal pig model of chronic myocardial infarction.^96^ More recently, 2 studies have focused on the repair of neurological injury using mouse models of neurogenic pain^109^ and spinal cord injury.^110^ A recent highly translational study using healthy non-human primates sought to understand the distribution and safety profile of intravenous administration of 2 types of MSCs—BM and human ESCs—cultured as 3D spheroids.^35^ Collectively, the increasing numbers of *in vivo* disease model studies and large animals demonstrate the strong interest and potential of 3D MSC spheroids for clinical use in a broad range of disease indications.

## Conclusions and Future Considerations

**Figure 2.**
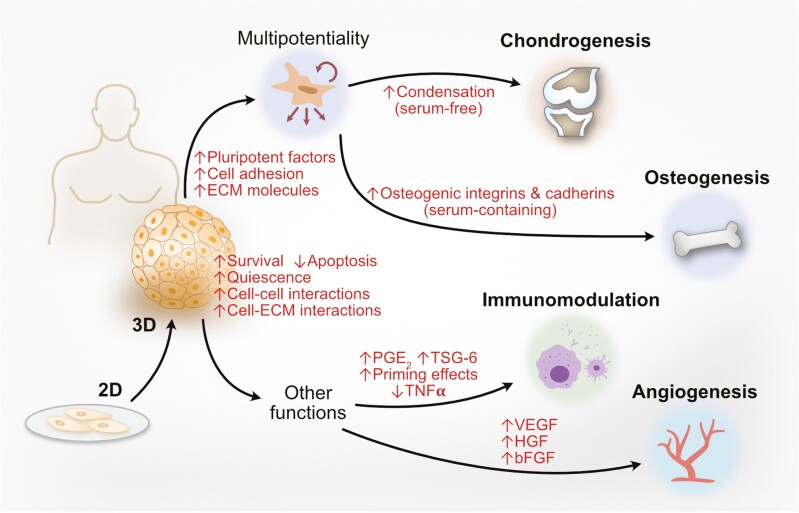
3-Dimensional (3D) multicellular spheroid culture of human mesenchymal stem cells (MSCs) induce profound biological changes. Compared to 2D monolayer-cultured cells, 3D spheroid MSCs demonstrate improved cell viability/survival, decreased apoptosis, and increased cellular quiescence. Cell-cell and cell-extracellular matrix (ECM) interactions are increased as well. In terms of multilineage/multipotential differentiation capacity, MSC chondrogenesis requires 3D serum-free conditions to increase condensation, whereas 3D MSC spheroid culture under typical serum-containing conditions increase pluripotency factor expression as well cell adhesion and ECM proteins, especially for osteogenesis. Immunomodulation and angiogenic/wound healing functions are improved as well in 3D MSC spheroids, largely through the increased expression of many paracrine factors. PGE_2_, prostaglandin E_2_; TSG6, tumor necrosis factor-stimulated gene 6; TNFα, tumor necrosis factor-α; VEGF, vascular endothelial growth factor; HGF, hepatocyte growth factor; bFGF, basic fibroblast growth factor.

The increasing numbers of accumulated reports strongly support that 3D spheroid culture for MSCs has therapeutically useful outcomes, and may also recapitulate many aspects of in vivo MSC biology (Fig. 2). As exciting and interesting as the results are from 3D MSC spheroid culture, one of the biggest concerns may be the methodology itself: culture methods in 3D are by nature more complex than 2D monolayer culture, with more room to adjust existing parameters as well as add new ones.^111^ Even when the 3D in vitro cell culture methodology is limited to multicellular spheroids, emerging data is showing that there are profound metabolic and proliferative/survival differences between cells within different locations of the spheroid^112,113^ (and recently reviewed in^114^); even cell size is altered, which in addition to obviously affecting biophysical parameters, appears to also have a translational impact.^115^ In addition, tissue-specific functional propensity of MSCs cultured in standard 2D conditions has emerged after decades of accumulated reports.^26^ While the relatively lower numbers of 3D-cultured MSC spheroid reports make it difficult currently to discern tissue-specific differences in functional outcome, this important MSC-specific variability should be evaluated in future 3D studies as clinical efficacy may be implicated. Clearly, rigorous examination and execution of in vitro 3D culture conditions are critical for broad use of such innovative methods. Recent advances in profiling technologies—both at the gene expression as well as protein level—are already providing more precise and granular information into the broad and profound changes brought about by the 3D spheroid culture of MSCs.^116,117^ It is anticipated in this rapidly advancing field that such tools and other novel technologies will continue to yield important data revealing how nuanced changes in 3D culture methodology can shape MSC biology for a better understanding of its original in vivo niche, as well as improve therapeutic outcome after *ex vivo* expansion.

## Supplementary Material

szad011_suppl_Supplementary_Material

## Data Availability

No new data were generated or analyzed in support of this research.

## References

[1] Benya PD , Shaffer JD. Dedifferentiated chondrocytes reexpress the differentiated collagen phenotype when cultured in agarose gels. Cell. 1982;30(1):215-224. https://doi.org/10.1016/0092-8674(82)90027-7 7127471

[2] Risau W , Sariola H, Zerwes HG, et al. Vasculogenesis and angiogenesis in embryonic-stem-cell-derived embryoid bodies. Development. 1988;102(3):471-478. https://doi.org/10.1242/dev.102.3.471 2460305

[3] Baker BM , Chen CS. Deconstructing the third dimension: how 3D culture microenvironments alter cellular cues. J Cell Sci. 2012;125(Pt 13):3015-3024. https://doi.org/10.1242/jcs.079509 22797912 PMC3434846

[4] O’Brien LE , Zegers MM, Mostov KE. Opinion: building epithelial architecture: insights from three-dimensional culture models. Nat Rev Mol Cell Biol. 2002;3(7):531-537. https://doi.org/10.1038/nrm859 12094219

[5] Sandberg R , Ernberg I. The molecular portrait of in vitro growth by meta-analysis of gene-expression profiles. Genome Biol. 2005;6(8):R65. https://doi.org/10.1186/gb-2005-6-8-r65 16086847 PMC1273632

[6] Lopes-Ramos CM , Paulson JN, Chen CY, et al. Regulatory network changes between cell lines and their tissues of origin. BMC Genom. 2017;18(1):723. https://doi.org/10.1186/s12864-017-4111-x PMC5596945 28899340

[7] Baker DE , Harrison NJ, Maltby E, et al. Adaptation to culture of human embryonic stem cells and oncogenesis in vivo. Nat Biotechnol. 2007;25(2):207-215. https://doi.org/10.1038/nbt1285 17287758

[8] Zhu J , Adli M, Zou JY, et al. Genome-wide chromatin state transitions associated with developmental and environmental cues. Cell. 2013;152(3):642-654. https://doi.org/10.1016/j.cell.2012.12.033 23333102 PMC3563935

[9] Martin GR , Evans MJ. Differentiation of clonal lines of teratocarcinoma cells: formation of embryoid bodies in vitro. Proc Natl Acad Sci USA. 1975;72(4):1441-1445. https://doi.org/10.1073/pnas.72.4.1441 1055416 PMC432551

[10] Martin GR. Isolation of a pluripotent cell line from early mouse embryos cultured in medium conditioned by teratocarcinoma stem cells. Proc Natl Acad Sci USA. 1981;78(12):7634-7638. https://doi.org/10.1073/pnas.78.12.7634 6950406 PMC349323

[11] Takahashi K , Yamanaka S. Induction of pluripotent stem cells from mouse embryonic and adult fibroblast cultures by defined factors. Cell. 2006;126(4):663-676. https://doi.org/10.1016/j.cell.2006.07.024 16904174

[12] Takahashi K , Tanabe K, Ohnuki M, et al. Induction of pluripotent stem cells from adult human fibroblasts by defined factors. Cell. 2007;131(5):861-872. https://doi.org/10.1016/j.cell.2007.11.019 18035408

[13] Itskovitz-Eldor J , Schuldiner M, Karsenti D, et al. Differentiation of human embryonic stem cells into embryoid bodies compromising the three embryonic germ layers. Mol Med. 2000;6(2):88-95. https://doi.org/10.1007/bf03401776 10859025 PMC1949933

[14] Shamblott MJ , Axelman J, Wang S, et al. Derivation of pluripotent stem cells from cultured human primordial germ cells. Proc Natl Acad Sci USA. 1998;95(23):13726-13731. https://doi.org/10.1073/pnas.95.23.13726 9811868 PMC24887

[15] Hyun I , Taylor P, Testa G, et al. Ethical standards for human-to-animal chimera experiments in stem cell research. Cell Stem Cell. 2007;1(2):159-163. https://doi.org/10.1016/j.stem.2007.07.015 18383627

[16] Jaenisch R , Young R. Stem cells, the molecular circuitry of pluripotency and nuclear reprogramming. Cell. 2008;132(4):567-582. https://doi.org/10.1016/j.cell.2008.01.015 18295576 PMC4142810

[17] Reynolds BA , Weiss S. Generation of neurons and astrocytes from isolated cells of the adult mammalian central nervous system. Science. 1992;255(5052):1707-1710. https://doi.org/10.1126/science.1553558 1553558

[18] Dontu G , Abdallah WM, Foley JM, et al. In vitro propagation and transcriptional profiling of human mammary stem/progenitor cells. Genes Dev. 2003;17(10):1253-1270. https://doi.org/10.1101/gad.1061803 12756227 PMC196056

[19] Singh SK , Clarke ID, Terasaki M, et al. Identification of a cancer stem cell in human brain tumors. Cancer Res. 2003;63(18):5821-5828. 14522905

[20] Ponti D , Costa A, Zaffaroni N, et al. Isolation and in vitro propagation of tumorigenic breast cancer cells with stem/progenitor cell properties. Cancer Res. 2005;65(13):5506-5511. https://doi.org/10.1158/0008-5472.CAN-05-0626 15994920

[21] Todaro M , Alea MP, Di Stefano AB, et al. Colon cancer stem cells dictate tumor growth and resist cell death by production of interleukin-4. Cell Stem Cell. 2007;1(4):389-402. https://doi.org/10.1016/j.stem.2007.08.001 18371377

[22] Eramo A , Lotti F, Sette G, et al. Identification and expansion of the tumorigenic lung cancer stem cell population. Cell Death Differ. 2008;15(3):504-514. https://doi.org/10.1038/sj.cdd.4402283 18049477

[23] Quintana E , Shackleton M, Sabel MS, et al. Efficient tumour formation by single human melanoma cells. Nature. 2008;456(7222):593-598. https://doi.org/10.1038/nature07567 19052619 PMC2597380

[24] Shmelkov SV , Butler JM, Hooper AT, et al. CD133 expression is not restricted to stem cells, and both CD133+ and CD133- metastatic colon cancer cells initiate tumors. J Clin Invest. 2008;118(6):2111-2120. https://doi.org/10.1172/JCI34401 18497886 PMC2391278

[25] Kaiser J. The cancer stem cell gamble. Science. 2015;347(6219):226-229. https://doi.org/10.1126/science.347.6219.226 25593170

[26] Yen BL , Liu KJ, Sytwu HK, et al. Clinical implications of differential functional capacity between tissue-specific human mesenchymal stromal/stem cells. FEBS J. 2022. 10.1111/febs.16438 35303395

[27] Dominici M , Le Blanc K, Mueller I, et al. Minimal criteria for defining multipotent mesenchymal stromal cells. The International Society for Cellular Therapy position statement. Cytotherapy. 2006;8(4):315-317. https://doi.org/10.1080/14653240600855905 16923606

[28] Arala-Chaves MP , Hope L, Korn JH, Fudenberg H. Role of adherent cells in immune responses to phytohemagglutinin and concanavalin A. Eur J Immunol. 1978;8(2):77-81. https://doi.org/10.1002/eji.1830080202 306923

[29] Triglia T , Burns GF, Werkmeister JA. Rapid changes in surface antigen expression by blood monocytes cultured in suspension or adherent to plastic. Blood. 1985;65(4):921-928. 3978234

[30] Habanjar O , Diab-Assaf M, Caldefie-Chezet F, et al. 3D cell culture systems: tumor application, advantages, and disadvantages. Int J Mol Sci. 2021;22(22):12200-12234. 10.3390/ijms222212200 34830082 PMC8618305

[31] Williams DF. There is no such thing as a biocompatible material. Biomaterials. 2014;35(38):10009-10014. https://doi.org/10.1016/j.biomaterials.2014.08.035 25263686

[32] Cosgrove BD , Mui KL, Driscoll TP, et al. N-cadherin adhesive interactions modulate matrix mechanosensing and fate commitment of mesenchymal stem cells. Nat Mater. 2016;15(12):1297-1306. https://doi.org/10.1038/nmat4725 27525568 PMC5121068

[33] Alimperti S , You H, George T, Agarwal SK, Andreadis ST. Cadherin-11 regulates both mesenchymal stem cell differentiation into smooth muscle cells and the development of contractile function in vivo. J Cell Sci. 2014;127(Pt 12):2627-2638. https://doi.org/10.1242/jcs.134833 24741067 PMC4058109

[34] Alimperti S , Andreadis ST. CDH2 and CDH11 act as regulators of stem cell fate decisions. Stem Cell Res. 2015;14(3):270-282. https://doi.org/10.1016/j.scr.2015.02.002 25771201 PMC4439315

[35] Yeung CK , Yan YP, Yan L, et al. Preclinical safety evaluation and tracing of human mesenchymal stromal cell spheroids following intravenous injection into cynomolgus monkeys. Biomaterials. 2022;289:121759. https://doi.org/10.1016/j.biomaterials.2022.121759 36075143

[36] Nombela-Arrieta C , Ritz J, Silberstein LE. The elusive nature and function of mesenchymal stem cells. Nat Rev Mol Cell Biol. 2011;12(2):126-131. https://doi.org/10.1038/nrm3049 21253000 PMC3346289

[37] Potapova IA , Gaudette GR, Brink PR, et al. Mesenchymal stem cells support migration, extracellular matrix invasion, proliferation, and survival of endothelial cells in vitro. Stem Cells. 2007;25(7):1761-1768. https://doi.org/10.1634/stemcells.2007-0022 17395769

[38] Bohrnsen F , Lindner U, Meier M, et al. Murine mesenchymal progenitor cells from different tissues differentiated via mesenchymal microspheres into the mesodermal direction. BMC Cell Biol. 2009;10:92. https://doi.org/10.1186/1471-2121-10-92 20021685 PMC2809059

[39] Kapur SK , Wang X, Shang H, et al. Human adipose stem cells maintain proliferative, synthetic and multipotential properties when suspension cultured as self-assembling spheroids. Biofabrication. 2012;4(2):025004. https://doi.org/10.1088/1758-5082/4/2/025004 22522924 PMC3401583

[40] Mackay AM , Beck SC, Murphy JM, et al. Chondrogenic differentiation of cultured human mesenchymal stem cells from marrow. Tissue Eng. 1998;4(4):415-428. https://doi.org/10.1089/ten.1998.4.415 9916173

[41] Fennema E , Rivron N, Rouwkema J, van Blitterswijk C, de Boer J. Spheroid culture as a tool for creating 3D complex tissues. Trends Biotechnol. 2013;31(2):108-115. https://doi.org/10.1016/j.tibtech.2012.12.003 23336996

[42] Kale S , Biermann S, Edwards C, et al. Three-dimensional cellular development is essential for ex vivo formation of human bone. Nat Biotechnol. 2000;18(9):954-958. https://doi.org/10.1038/79439 10973215

[43] Chakradhar S. Put to the test: organoid-based testing becomes a clinical tool. Nat Med. 2017;23(7):796-799. https://doi.org/10.1038/nm0717-796 28697178

[44] Engler AJ , Sen S, Sweeney HL, Discher DE. Matrix elasticity directs stem cell lineage specification. Cell. 2006;126(4):677-689. https://doi.org/10.1016/j.cell.2006.06.044 16923388

[45] Dupont S , Morsut L, Aragona M, et al. Role of YAP/TAZ in mechanotransduction. Nature. 2011;474(7350):179-183. https://doi.org/10.1038/nature10137 21654799

[46] Limoli CL , Rola R, Giedzinski E, et al. Cell-density-dependent regulation of neural precursor cell function. Proc Natl Acad Sci USA. 2004;101(45):16052-16057. https://doi.org/10.1073/pnas.0407065101 15522966 PMC528770

[47] Ma Q , Wang Y, Lo AS, Gomes EM, Junghans RP. Cell density plays a critical role in ex vivo expansion of T cells for adoptive immunotherapy. J Biomed Biotechnol. 2010;2010:386545. https://doi.org/10.1155/2010/386545 20625484 PMC2896674

[48] Pirkmajer S , Chibalin AV. Serum starvation: caveat emptor. Am J Physiol Cell Physiol. 2011;301(2):C272-C279. https://doi.org/10.1152/ajpcell.00091.2011 21613612

[49] Wolock SL , Krishnan I, Tenen DE, et al. Mapping distinct bone marrow niche populations and their differentiation paths. Cell Rep. 2019;28(2):302-311.e5. https://doi.org/10.1016/j.celrep.2019.06.031 31291568 PMC6684313

[50] Baryawno N , Przybylski D, Kowalczyk MS, et al. A cellular taxonomy of the bone marrow stroma in homeostasis and leukemia. Cell. 2019;177(7):1915-1932.e16. https://doi.org/10.1016/j.cell.2019.04.040 31130381 PMC6570562

[51] Helbling PM , Pineiro-Yanez E, Gerosa R, et al. Global transcriptomic profiling of the bone marrow stromal microenvironment during postnatal development, aging, and inflammation. Cell Rep. 2019;29(10):3313-3330.e4. https://doi.org/10.1016/j.celrep.2019.11.004 31801092

[52] Tacchetti C , Tavella S, Dozin B, et al. Cell condensation in chondrogenic differentiation. Exp Cell Res. 1992;200(1):26-33. https://doi.org/10.1016/s0014-4827(05)80067-9 1563490

[53] Hall BK , Miyake T. All for one and one for all: condensations and the initiation of skeletal development. Bioessays. 2000;22(2):138-147. https://doi.org/10.1002/(SICI)1521-1878(200002)22:2<138::AID-BIES5>3.0.CO;2-4 10655033

[54] Ahmed N , Iu J, Brown CE, et al. Serum- and growth-factor-free three-dimensional culture system supports cartilage tissue formation by promoting collagen synthesis via Sox9-Col2a1 interaction. Tissue Eng Part A. 2014;20(15-16):2224-2233. 24606204 10.1089/ten.tea.2013.0559 PMC4137332

[55] Guo W , Keckesova Z, Donaher JL, et al. Slug and Sox9 cooperatively determine the mammary stem cell state. Cell. 2012;148(5):1015-1028. https://doi.org/10.1016/j.cell.2012.02.008 22385965 PMC3305806

[56] Hsieh CC , Yen BL, Chang CC, et al. Wnt antagonism without TGFbeta induces rapid MSC chondrogenesis via increasing AJ interactions and restricting lineage commitment. iScience. 2023;26(1):105713. https://doi.org/10.1016/j.isci.2022.105713 36582823 PMC9792887

[57] Huang GS , Dai LG, Yen BL, Hsu S-H. Spheroid formation of mesenchymal stem cells on chitosan and chitosan-hyaluronan membranes. Biomaterials. 2011;32(29):6929-6945. https://doi.org/10.1016/j.biomaterials.2011.05.092 21762982

[58] Cheng NC , Wang S, Young TH. The influence of spheroid formation of human adipose-derived stem cells on chitosan films on sternness and differentiation capabilities. Biomaterials. 2012;33(6):1748-1758. https://doi.org/10.1016/j.biomaterials.2011.11.049 22153870

[59] Baraniak PR , McDevitt TC. Scaffold-free culture of mesenchymal stem cell spheroids in suspension preserves multilineage potential. Cell Tissue Res. 2012;347(3):701-711. https://doi.org/10.1007/s00441-011-1215-5 21833761 PMC4149251

[60] Lengner CJ , Camargo FD, Hochedlinger K, et al. Oct4 expression is not required for mouse somatic stem cell self-renewal. Cell Stem Cell. 2007;1(4):403-415. https://doi.org/10.1016/j.stem.2007.07.020 18159219 PMC2151746

[61] Tsai CC , Su PF, Huang YF, Yew T-L, Hung S-C. Oct4 and Nanog directly regulate Dnmt1 to maintain self-renewal and undifferentiated state in mesenchymal stem cells. Mol Cell. 2012;47(2):169-182. https://doi.org/10.1016/j.molcel.2012.06.020 22795133

[62] Piazzolla D , Palla AR, Pantoja C, et al. Lineage-restricted function of the pluripotency factor NANOG in stratified epithelia. Nat Commun. 2014;5:4226. https://doi.org/10.1038/ncomms5226 24979572

[63] Frith JE , Thomson B, Genever PG. Dynamic three-dimensional culture methods enhance mesenchymal stem cell properties and increase therapeutic potential. Tissue Eng Part C-Methods. 2010;16(4):735-749. 19811095 10.1089/ten.TEC.2009.0432

[64] Wang WJ , Itaka K, Ohba S, et al. 3D spheroid culture system on micropatterned substrates for improved differentiation efficiency of multipotent mesenchymal stem cells. Biomaterials. 2009;30(14):2705-2715. https://doi.org/10.1016/j.biomaterials.2009.01.030 19215979

[65] Suenaga H , Furukawa KS, Suzuki Y, et al. Bone regeneration in calvarial defects in a rat model by implantation of human bone marrow-derived mesenchymal stromal cell spheroids. J Mater Sci Mater Med. 2015;26(11):254. 10.1007/s10856-015-5591-3 26449444 PMC4598349

[66] Griffin FE , Schiavi J, McDevitt TC, McGarry JP, McNamara LM. The role of adhesion junctions in the biomechanical behaviour and osteogenic differentiation of 3D mesenchymal stem cell spheroids. J Biomech. 2017;59:71-79. https://doi.org/10.1016/j.jbiomech.2017.05.014 28577903 PMC6392184

[67] Murphy KC , Hoch AI, Harvestine JN, Zhou D, Leach JK. Mesenchymal stem cell spheroids retain osteogenic phenotype through alpha2beta1 signaling. Stem Cells Transl Med. 2016;5(9):1229-1237. https://doi.org/10.5966/sctm.2015-0412 27365484 PMC4996446

[68] Kim J , Adachi T. Cell-fate decision of mesenchymal stem cells toward osteocyte differentiation is committed by spheroid culture. Sci Rep. 2021;11(1):13204. https://doi.org/10.1038/s41598-021-92607-z 34168224 PMC8225633

[69] Kittaka M , Kajiya M, Shiba H, et al. Clumps of a mesenchymal stromal cell/extracellular matrix complex can be a novel tissue engineering therapy for bone regeneration. Cytotherapy. 2015;17(7):860-873. https://doi.org/10.1016/j.jcyt.2015.01.007 25743634

[70] Yamaguchi Y , Ohno J, Sato A, Kido H, Fukushima T. Mesenchymal stem cell spheroids exhibit enhanced in-vitro and in-vivo osteoregenerative potential. BMC Biotechnol. 2014;14:105. https://doi.org/10.1186/s12896-014-0105-9 25479895 PMC4299781

[71] McBeath R , Pirone DM, Nelson CM, Bhadriraju K, Chen CS. Cell shape, cytoskeletal tension, and RhoA regulate stem cell lineage commitment. Dev Cell. 2004;6(4):483-495. https://doi.org/10.1016/s1534-5807(04)00075-9 15068789

[72] Tseng PC , Young TH, Wang TM, et al. Spontaneous osteogenesis of MSCs cultured on 3D microcarriers through alteration of cytoskeletal tension. Biomaterials. 2012;33(2):556-564. https://doi.org/10.1016/j.biomaterials.2011.09.090 22024363

[73] Ahmed TA , Hincke MT. Mesenchymal stem cell-based tissue engineering strategies for repair of articular cartilage. Histol Histopathol. 2014;29(6):669-689. https://doi.org/10.14670/HH-29.669 24452855

[74] Amos PJ , Kapur SK, Stapor PC, et al. Human adipose-derived stromal cells accelerate diabetic wound healing: impact of cell formulation and delivery. Tissue Eng Part A. 2010;16(5):1595-1606. https://doi.org/10.1089/ten.TEA.2009.0616 20038211 PMC2952117

[75] Xu Y , Shi T, Xu A, Zhang L. 3D spheroid culture enhances survival and therapeutic capacities of MSCs injected into ischemic kidney. J Cell Mol Med. 2016;20(7):1203-1213. https://doi.org/10.1111/jcmm.12651 26914637 PMC4929304

[76] Cesarz Z , Funnell JL, Guan J, Tamama K. Soft elasticity-associated signaling and bone morphogenic protein 2 are key regulators of mesenchymal stem cell spheroidal aggregates. Stem Cells Dev. 2016;25(8):622-635. https://doi.org/10.1089/scd.2015.0356 26916040 PMC4834581

[77] Jiang B , Yan L, Miao Z, et al. Spheroidal formation preserves human stem cells for prolonged time under ambient conditions for facile storage and transportation. Biomaterials. 2017;133:275-286. https://doi.org/10.1016/j.biomaterials.2017.03.050 28460350

[78] Guo L , Zhou Y, Wang S, Wu Y. Epigenetic changes of mesenchymal stem cells in three-dimensional (3D) spheroids. J Cell Mol Med. 2014;18(10):2009-2019. https://doi.org/10.1111/jcmm.12336 25090911 PMC4244016

[79] Bhang SH , Cho SW, La WG, et al. Angiogenesis in ischemic tissue produced by spheroid grafting of human adipose-derived stromal cells. Biomaterials. 2011;32(11):2734-2747. https://doi.org/10.1016/j.biomaterials.2010.12.035 21262528

[80] Murphy KC , Fang SY, Leach JK. Human mesenchymal stem cell spheroids in fibrin hydrogels exhibit improved cell survival and potential for bone healing. Cell Tissue Res. 2014;357(1):91-99. https://doi.org/10.1007/s00441-014-1830-z 24781147 PMC4077909

[81] Bartosh TJ , Ylostalo JH, Mohammadipoor A, et al. Aggregation of human mesenchymal stromal cells (MSCs) into 3D spheroids enhances their antiinflammatory properties. Proc Natl Acad Sci USA. 2010;107(31):13724-13729. 20643923 10.1073/pnas.1008117107 PMC2922230

[82] Hsu SH , Huang TB, Cheng SJ, et al. Chondrogenesis from human placenta-derived mesenchymal stem cells in three-dimensional scaffolds for cartilage tissue engineering. Tissue Eng Part A. 2011;17(11-12):1549-1560. 21284540 10.1089/ten.TEA.2010.0419

[83] Wang X , Jiang B, Sun H, et al. Noninvasive application of mesenchymal stem cell spheres derived from hESC accelerates wound healing in a CXCL12-CXCR4 axis-dependent manner. Theranostics. 2019;9(21):6112-6128. https://doi.org/10.7150/thno.32982 31534540 PMC6735514

[84] Bhang SH , Lee S, Shin JY, et al. Transplantation of cord blood mesenchymal stem cells as spheroids enhances vascularization. Tissue Eng Part A. 2012;18(19-20):2138-2147. 22559333 10.1089/ten.tea.2011.0640 PMC3463282

[85] Wang LT , Liu KJ, Sytwu HK, Yen M-L, Yen BL. Advances in mesenchymal stem cell therapy for immune and inflammatory diseases: use of cell-free products and human pluripotent stem cell-derived mesenchymal stem cells. Stem Cells Transl Med. 2021;10(9):1288-1303. https://doi.org/10.1002/sctm.21-0021 34008922 PMC8380447

[86] Bartosh TJ , Ylostalo JH, Bazhanov N, Kuhlman J, Prockop DJ. Dynamic compaction of human mesenchymal stem/precursor cells into spheres self-activates caspase-dependent IL1 signaling to enhance secretion of modulators of inflammation and immunity (PGE2, TSG6, and STC1). Stem Cells. 2013;31(11):2443-2456. https://doi.org/10.1002/stem.1499 23922312 PMC3834191

[87] Petrenko Y , Sykova E, Kubinova S. The therapeutic potential of three-dimensional multipotent mesenchymal stromal cell spheroids. Stem Cell Res Ther. 2017;8(1):94. https://doi.org/10.1186/s13287-017-0558-6 28446248 PMC5406927

[88] Ohori-Morita Y , Niibe K, Limraksasin P, et al. Novel mesenchymal stem cell spheroids with enhanced stem cell characteristics and bone regeneration ability. Stem Cells Transl Med. 2022;11(4):434-449. https://doi.org/10.1093/stcltm/szab030 35267026 PMC9052431

[89] Zimmermann JA , McDevitt TC. Pre-conditioning mesenchymal stromal cell spheroids for immunomodulatory paracrine factor secretion. Cytotherapy. 2014;16(3):331-345. https://doi.org/10.1016/j.jcyt.2013.09.004 24219905

[90] Zimmermann JA , Hettiaratchi MH, McDevitt TC. Enhanced immunosuppression of T cells by sustained presentation of bioactive interferon-gamma within three-dimensional mesenchymal stem cell constructs. Stem Cells Transl Med. 2017;6(1):223-237. https://doi.org/10.5966/sctm.2016-0044 28170190 PMC5442746

[91] Redondo-Castro E , Cunningham CJ, Miller J, et al. Changes in the secretome of tri-dimensional spheroid-cultured human mesenchymal stem cells in vitro by interleukin-1 priming. Stem Cell Res Ther. 2018;9. 10.1186/s13287-017-0753-5 PMC5773162 29343288

[92] Miranda JP , Camoes SP, Gaspar MM, et al. The secretome derived from 3D-cultured umbilical cord tissue MSCs counteracts manifestations typifying rheumatoid arthritis. Front Immunol. 2019;10:18. https://doi.org/10.3389/fimmu.2019.00018 30804924 PMC6370626

[93] Jiang B , Fu X, Yan L, et al. Transplantation of human ESC-derived mesenchymal stem cell spheroids ameliorates spontaneous osteoarthritis in rhesus macaques. Theranostics. 2019;9(22):6587-6600. https://doi.org/10.7150/thno.35391 31588237 PMC6771254

[94] Potapova IA , Brink PR, Cohen IS, Doronin SV. Culturing of human mesenchymal stem cells as three-dimensional aggregates induces functional expression of CXCR4 that regulates adhesion to endothelial cells. J Biol Chem. 2008;283(19):13100-13107. https://doi.org/10.1074/jbc.M800184200 18334485 PMC2442325

[95] Santos JM , Camoes SP, Filipe E, et al. Three-dimensional spheroid cell culture of umbilical cord tissue-derived mesenchymal stromal cells leads to enhanced paracrine induction of wound healing. Stem Cell Res Ther. 2015;6(1):90. https://doi.org/10.1186/s13287-015-0082-5 25956381 PMC4448539

[96] Emmert MY , Wolint P, Winklhofer S, et al. Transcatheter based electromechanical mapping guided intramyocardial transplantation and in vivo tracking of human stem cell based three dimensional microtissues in the porcine heart. Biomaterials. 2013;34(10):2428-2441. https://doi.org/10.1016/j.biomaterials.2012.12.021 23332174

[97] Semenza GL. HIF-1: using two hands to flip the angiogenic switch. Cancer Metastasis Rev. 2000;19(1-2):59-65. https://doi.org/10.1023/a:1026544214667 11191064

[98] Watt FM , Huck WT. Role of the extracellular matrix in regulating stem cell fate. Nat Rev Mol Cell Biol. 2013;14(8):467-473. https://doi.org/10.1038/nrm3620 23839578

[99] Brindo da Cruz IC , Velosa APP, Carrasco S, et al. Post-adipose-derived stem cells (ADSC) stimulated by collagen type V (Col V) mitigate the progression of osteoarthritic rabbit articular cartilage. Front Cell Dev Biol. 2021;9:606890. https://doi.org/10.3389/fcell.2021.606890 33829012 PMC8019831

[100] Mruthyunjaya S , Manchanda R, Godbole R, et al. Laminin-1 induces neurite outgrowth in human mesenchymal stem cells in serum/differentiation factors-free conditions through activation of FAK-MEK/ERK signaling pathways. Biochem Biophys Res Commun. 2010;391(1):43-48. https://doi.org/10.1016/j.bbrc.2009.10.158 19895795

[101] Nakamura R , Nakamura F, Fukunaga S. Contrasting effect of perlecan on adipogenic and osteogenic differentiation of mesenchymal stem cells in vitro. Anim Sci J. 2014;85(3):262-270. https://doi.org/10.1111/asj.12116 24000897

[102] Hoefner C , Muhr C, Horder H, et al. Human adipose-derived mesenchymal stromal/stem cell spheroids possess high adipogenic capacity and acquire an adipose tissue-like extracellular matrix pattern. Tissue Eng Part A. 2020;26(15-16):915-926. 32070231 10.1089/ten.TEA.2019.0206

[103] Moerman EJ , Teng K, Lipschitz DA, Lecka-Czernik B. Aging activates adipogenic and suppresses osteogenic programs in mesenchymal marrow stroma/stem cells: the role of PPAR-gamma2 transcription factor and TGF-beta/BMP signaling pathways. Aging Cell. 2004;3(6):379-389. https://doi.org/10.1111/j.1474-9728.2004.00127.x 15569355 PMC1850101

[104] Moritani Y , Usui M, Sano K, et al. Spheroid culture enhances osteogenic potential of periodontal ligament mesenchymal stem cells. J Periodontal Res. 2018;53(5):870-882. https://doi.org/10.1111/jre.12577 29900548

[105] Imamura A , Kajiya H, Fujisaki S, et al. Three-dimensional spheroids of mesenchymal stem/stromal cells promote osteogenesis by activating stemness and Wnt/beta-catenin. Biochem Biophys Res Commun. 2020;523(2):458-464. https://doi.org/10.1016/j.bbrc.2019.12.066 31882121

[106] Muttigi MS , Kim BJ, Kumar H, et al. Efficacy of matrilin-3-primed adipose-derived mesenchymal stem cell spheroids in a rabbit model of disc degeneration. Stem Cell Res Ther. 2020;11(1):363. https://doi.org/10.1186/s13287-020-01862-w 32831130 PMC7444036

[107] Shimazawa Y , Kusamori K, Tsujimura M, et al. Intravenous injection of mesenchymal stem cell spheroids improves the pulmonary delivery and prolongs in vivo survival. Biotechnol J. 2022;17(1):e2100137. https://doi.org/10.1002/biot.202100137 34581003

[108] Park IS , Rhie JW, Kim SH. A novel three-dimensional adipose-derived stem cell cluster for vascular regeneration in ischemic tissue. Cytotherapy. 2014;16(4):508-522. https://doi.org/10.1016/j.jcyt.2013.08.011 24210783

[109] Lee N , Park GT, Lim JK, et al. Mesenchymal stem cell spheroids alleviate neuropathic pain by modulating chronic inflammatory response genes. Front Immunol. 2022;13:940258. https://doi.org/10.3389/fimmu.2022.940258 36003384 PMC9393760

[110] Deng J , Li M, Meng F, et al. 3D spheroids of human placenta-derived mesenchymal stem cells attenuate spinal cord injury in mice. Cell Death Dis. 2021;12(12):1096. 10.1038/s41419-021-04398-w 34803160 PMC8606575

[111] Ezquerra S , Zuleta A, Arancibia R, et al. Functional properties of human-derived mesenchymal stem cell spheroids: a meta-analysis and systematic review. Stem Cells Int. 2021;2021:8825332. https://doi.org/10.1155/2021/8825332 33884001 PMC8041538

[112] Coyle R , Yao J, Richards D, Mei Y. The effects of metabolic substrate availability on human adipose-derived stem cell spheroid survival. Tissue Eng Part A. 2019;25(7-8):620-631. https://doi.org/10.1089/ten.TEA.2018.0163 30226442 PMC6482921

[113] Schmitz C , Potekhina E, Belousov VV, et al. Hypoxia onset in mesenchymal stem cell spheroids: monitoring with hypoxia reporter cells. Front Bioeng Biotechnol. 2021;9:611837. 10.3389/fbioe.2019.00292 33614611 PMC7892969

[114] Kouroupis D , Correa D. Increased mesenchymal stem cell functionalization in three-dimensional manufacturing settings for enhanced therapeutic applications. Front Bioeng Biotechnol. 2021;9:621748. https://doi.org/10.3389/fbioe.2021.621748 33644016 PMC7907607

[115] Tietze S , Krater M, Jacobi A, et al. Spheroid culture of mesenchymal stromal cells results in morphorheological properties appropriate for improved microcirculation (vol 6, 1802104, 2019). Adv Sci. 2019;6(8):1802104. https://doi.org/10.1002/advs.201802104 PMC6469243 31016116

[116] Gallo A , Cuscino N, Contino F, et al. Changes in the transcriptome profiles of human amnion-derived mesenchymal stromal/stem cells induced by three-dimensional culture: a potential priming strategy to improve their properties. Int J Mol Sci. 2022;23(2). 10.3390/ijms23020863 PMC8778321 35055049

[117] Kusuma GD , Li A, Zhu D, et al. Effect of 2D and 3D culture microenvironments on mesenchymal stem cell-derived extracellular vesicles potencies. Front Cell Dev Biol. 2022;10:819726. https://doi.org/10.3389/fcell.2022.819726 35237601 PMC8882622

[118] Somaiah C , Kumar A, Mawrie D, et al. Collagen promotes higher adhesion, survival and proliferation of mesenchymal stem cells. PLoS One. 2015;10(12):e0145068e0145068. https://doi.org/10.1371/journal.pone.0145068 26661657 PMC4678765

[119] Smeriglio P , Lee J, Bhutani N. Soluble collagen VI treatment enhances mesenchymal stem cells expansion for engineering cartilage. Bioeng Transl Med. 2017;2(3):278-284. https://doi.org/10.1002/btm2.10078 29313037 PMC5689496

[120] Smaldone S , Clayton NP, del Solar M, et al. Fibrillin-1 regulates skeletal stem cell differentiation by modulating TGFbeta activity within the marrow niche. J Bone Miner Res. 2016;31(1):86-97. https://doi.org/10.1002/jbmr.2598 26189658 PMC5776390

[121] Redondo-Castro E , Cunningham CJ, Miller J, et al. Generation of human mesenchymal stem cell 3D spheroids using low-binding plates. Bio Protoc. 2018;8(16). 10.21769/BioProtoc.2968 PMC6173304 30294619

[122] Cimino M , Goncalves RM, Bauman E, et al. Optimization of the use of a pharmaceutical grade xeno-free medium for in vitro expansion of human mesenchymal stem/stromal cells. J Tissue Eng Regen Med. 2018;12(3):e1785-e1795. https://doi.org/10.1002/term.2588 29024519

[123] Cha BH , Kim JS, Bello A, et al. Efficient isolation and enrichment of mesenchymal stem cells from human embryonic stem cells by utilizing the interaction between integrin alpha5beta1 and fibronectin. Adv Sci. 2020;7(17):2001365. 10.1002/advs.202001365 PMC7507081 32995130

[124] Cho S , Choi H, Jeong H, et al. Preclinical study of human bone marrow-derived mesenchymal stem cells using a 3-dimensional manufacturing setting for enhancing spinal fusion. Stem Cells Transl Med. 2022;11(10):1072-1088. https://doi.org/10.1093/stcltm/szac052 36180050 PMC9585955

[125] Peng KY , Liu YH, Li YW, Yen BL, Yen M-L. Extracellular matrix protein laminin enhances mesenchymal stem cell (MSC) paracrine function through alphavbeta3/CD61 integrin to reduce cardiomyocyte apoptosis. J Cell Mol Med. 2017;21(8):1572-1583. https://doi.org/10.1111/jcmm.13087 28600799 PMC5543513

[126] Dong G , Wang S, Ge Y, et al. Serum-free culture system for spontaneous human mesenchymal stem cell spheroid formation. Stem Cells Int. 2019;2019:6041816. https://doi.org/10.1155/2019/6041816 31737076 PMC6815607

