# Supplementary material for: Three-Dimensional Spheroid Culture of Human Mesenchymal Stem Cells: Offering Therapeutic Advantages and In Vitro Glimpses of the In Vivo State
Source: Stem Cells Transl Med. 2023 May 15;12(5):235–44. doi: 10.1093/stcltm/szad011 (PMC10184701; doi:10.1093/stcltm/szad011)
Supplement: szad011_suppl_Supplementary_Material [file szad011_suppl_supplementary_material.docx]

**Table 1. Effects and expression of ECM molecules in 3D-cultured MSC spheroids (references in brackets).**

| **ECM molecules** | **Effects on MSCs** | **Expression in 3D-cultured spheroids** |
| --- | --- | --- |
| Collagen I | Promote adhesion, survival, and proliferation [118] | Upregulated [75] |
| Collagen IV | Upregulated during adipogenic induction [103] | Less expressed [103] |
| Collagen V | Enhance proliferation and chondrogenesis [100] | Highly expressed [95] |
| Collagen VI | Enhance expansion [119] | Highly expressed [95] |
| Fibrilin-1 | Fibrillin-1–deficiency induces bone loss and impair adipogenesis [120] | Expressed [121] |
| Fibronectin 1 | Selection/enrichment of MSC [122]  Improve adhesion, metabolic activity [123] | Upregulated [75]  Downregulated [124] |
| Laminin | Increase paracrine [125]  Induces neurite outgrowth [101] | Upregulated [75] |
| Perlecan | Promote osteogenesis, block adipogenesis [102] | Upregulated [126] |

**Table 2: 3D MSC spheroid-based *in vivo* models**

| **Functional outcome** | **Disease model** | **Animal used** | **MSC parameters** | | | **Reference** |
| --- | --- | --- | --- | --- | --- | --- |
|  |  |  | **Species** | **Tissue** | |  |
| Bone | Calvarial defect | Rat | Rat | | BM | 70 |
|  |  | Rat | Rat | | BM | 69 |
|  |  | Rat | Human | | BM | 65 |
|  |  | Mouse | Human | | Peridontal ligament | 105 |
|  |  | Mouse (aged) | Mouse | | BM | 107 |
|  | Femur fracture | Rat | Human | | BM | 88 |
| Intervertebral disc | Annular needle puncture | Rabbit | Human | | Adipose  (matrilin-3 primed) | 108 |
| Arthritis, rheumatoid | Adjuvant induced | Mouse | Human | | Secretome from umbilical cord | 92 |
| Arthrtis, osteo- | Spontaneous | Non-human primate | Human | | BM & ESC | J93 |
| Angiogenesis | Limb ischemia | Mouse | Human | | Adipose | 79 |
|  |  | Mouse | Human | | Umbilical cord blood | 84 |
|  |  | Mouse | Human | | Adipose | 110 |
| Wound healing | Excisional skin wound | Rat | Human | | Umbilical cord | 95 |
|  |  | Mouse | Human | | ESC | 83 |
|  |  | Mouse (diabetic db/db) | Human | | Adipose | 74 |
| Inflammation | Peritonitis | Mouse | Human | | BM | 81 |
|  | Colitis | Mouse | Human | | ESC | 77 |
|  | Pulmonary | Mouse | Mouse | | Adipose | 109 |
| Ischemia | Acute renal ischemia/ reperfusion injury | Rat | Human | | Adipose | 75 |
|  | Chronic myocardial infarction | Pig | Human | | Adipose | 96 |
| Neurogenic pain | Chronic constriction | Mouse | Human | | Tonsil | 112 |
| Spinal Cord | Hemi-section | Mouse | Human | | Placenta | 35 |
| Safety & distribution | Healthy | Non-human primate | Human | | ESC & Umbilical cord | 114 |

Abbreviations: BM, bone marrow; ESC, embryonic stem cell.
